# Jasmonates Coordinate Secondary with Primary Metabolism

**DOI:** 10.3390/metabo13091008

**Published:** 2023-09-13

**Authors:** Chen Luo, Jianfang Qiu, Yu Zhang, Mengya Li, Pei Liu

**Affiliations:** Department of Ecology, College of Resources and Environmental Sciences, China Agricultural University, Beijing 100193, China; b20173030224@cau.edu.cn (C.L.); qjf@cau.edu.cn (J.Q.); s20223030399@cau.edu.cn (Y.Z.)

**Keywords:** *cis*-jasmone, ecological interactions, growth–defense tradeoff, OPDA, volatile organic compounds

## Abstract

Jasmonates (JAs), including jasmonic acid (JA), its precursor 12-oxo-phytodienoic acid (OPDA) and its derivatives jasmonoyl-isoleucine (JA-Ile), methyl jasmonate (MeJA), *cis*-jasmone (CJ) and other oxylipins, are important in the regulation of a range of ecological interactions of plants with their abiotic and particularly their biotic environments. Plant secondary/specialized metabolites play critical roles in implementing these ecological functions of JAs. Pathway and transcriptional regulation analyses have established a central role of JA-Ile-mediated core signaling in promoting the biosynthesis of a great diversity of secondary metabolites. Here, we summarized the advances in JAs-induced secondary metabolites, particularly in secondary metabolites induced by OPDA and volatile organic compounds (VOCs) induced by CJ through signaling independent of JA-Ile. The roles of JAs in integrating and coordinating the primary and secondary metabolism, thereby orchestrating plant growth–defense tradeoffs, were highlighted and discussed. Finally, we provided perspectives on the improvement of the adaptability and resilience of plants to changing environments and the production of valuable phytochemicals by exploiting JAs-regulated secondary metabolites.

## 1. Introduction

Jasmonates (JAs) are a group of plant hormones/phytohormones, including jasmonic acid (JA) and related oxylipins, which are synthesized from polyunsaturated fatty acids, predominantly α-linolenic acid (α-LA), in plant cells [1,2]. Several excellent reviews have previously summarized the metabolism, signaling and transport of JAs [2,3,4,5,6,7,8,9,10,11]. Jasmonoyl-isoleucine (JA-Ile) is the major bioactive member of JAs; it activates the core JA signaling (also called COI1-dependent or JA-Ile signaling hereafter) by binding with its coreceptor Skp1-Cullin1-F-box-type (SCF) protein ubiquitin ligase complex SCF^COI1^-JAZ, leading to transcriptional reprogramming by the 26S proteasome-mediated degradation of the JAZ (JASMONATE-ZIM DOMAIN) transcriptional repressors [12,13,14,15]. In addition, the JA precursor *cis*-12-oxo-phytodienoic acid (OPDA) [16], volatile *cis*-jasmone (CJ) and hydroxylated JA (12-OH-JA) appear to have signaling independent of the JA-Ile-mediated core signaling pathway [1,10,17].

JA-Ile signaling plays primary roles in regulating numerous ecological interactions with biotic and abiotic environments, particularly in defenses against herbivories and necrotrophic pathogens [18,19], as well as in symbiotic interactions such as arbuscular mycorrhiza and root nodule symbiosis [20,21,22,23]. To coordinate ecological interactions with plant development and growth, JA-Ile signaling also mediates diverse developmental processes including seed germination; root growth and architecture; tuber and trichome formation; and flower development, in particular [3,24]. It is well accepted that secondary metabolites, in contrast to primary metabolites that are critical for the essential functions needed for plant survival, are currently well recognized as playing important roles in diverse ecological interactions with their biotic and abiotic environmental factors; thus, they are currently renamed as specialized metabolites due to their specialized functions (also called natural products). The control of secondary metabolic pathways in plants by transcription factors in the core JA signaling pathway has been previously reviewed [25,26,27,28]. We summarize the advances in plant secondary metabolites (PSMs) induced by JAs, with an emphasis on those secondary metabolites induced by OPDA and volatile organic compounds (VOCs) induced by CJ through signaling independent of JA-Ile. Furthermore, important roles of JAs in orchestrating plant growth and ecological interactions/defense by coupling the primary and secondary metabolism pathways are highlighted. Finally, the exploitation of JAs-regulated secondary metabolites in the improvement of the adaptability and resilience of plants/crops to changing environments and the production of high-value phytochemicals is discussed. 

## 2. Lineage-/Species-Dependent Induction of Diverse Secondary Metabolites by JA-Ile Signaling

As sessile organisms, plants have evolved sophisticated metabolic systems that produce a great diversity of metabolites in order to survive diverse and dynamic terrestrial environments. Using simple, inorganic precursors, a large variety of small-molecule organic compounds are synthesized; these have been classified by their actual or predicted functions into primary and secondary metabolites and plant hormones/phytohormones. While primary metabolites are essential for plant growth and development and are highly conserved, secondary metabolites are often lineage-specific and are implicated in ecological interactions between plants and their biotic and abiotic environments. Although increasing evidence shows that many secondary metabolites have a regulatory role, plant hormones are a particular group of secondary metabolites that play preeminent roles in regulating many processes in plant life including the synthesis of other metabolites by binding with their specific receptors [29].

Approximately 200,000 PSMs have been identified, and many more are expected to be discovered in the 391,000 described plant species. Based on their compositions and structures, PSMs can be classified into terpenoids, phenylpropanoids, nitrogen (N)- or/and sulfur (S)-containing compounds. PSMs have played vital roles in the evolution of plants [30], as illustrated by that cutin and phenolic compounds adapted the early plants to terrestrial environment by preventing plant cells from damage by ultraviolet (UV) light and desiccation [31]. Lignin is the most abundant polymer on earth derived from phenolic compounds, and it enables plants to grow bigger in stature, deal with gravity and develop vascular tissues for the movement of water [32,33,34]. The great diversity of PSMs in terms of chemical structures and spatial (cell, tissue, organ, species or taxa) and temporal (developmental stages or specific stimuli) production is critical for the adaptation of plants by involving them in diverse ecological interactions. 

**Figure 1:**
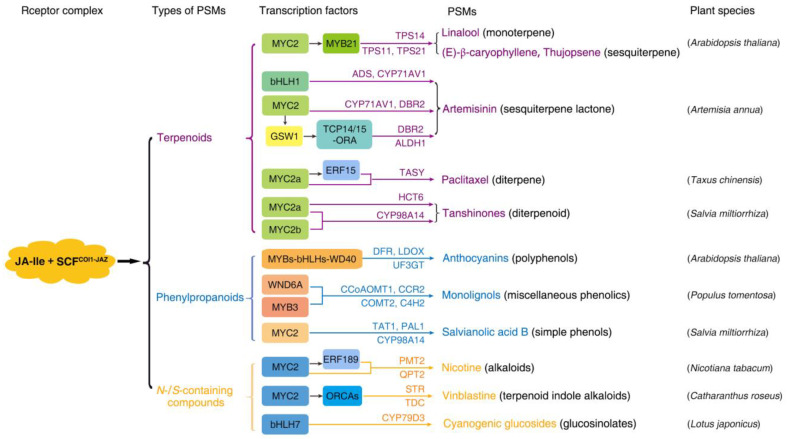
The chemical diversity and lineage/species specificity of PSMs induced by JA-Ile signaling. JA-Ile signaling is involved in the induction of all types of PSMs including terpenoids (purple), phenylpropanoids/phenolics (light blue) and N-/S-containing compounds (yellow) in a species-specific manner. The MYC2s act as the regulatory hub by themselves or synergistically with other TFs (indicated in the colored boxes) to promote the transcription of enzymes that catabolize the production of various PSMs, as indicated by the arrows, such as TPS (terpene synthase), ADS (amorpha-4,11-diene synthase), CYP71AV1 (cytochrome P450 monooxygenase 71AV1), DBR2 (double bond reductase 2), HCT6 (hydroxy-cinnamoyl transferase 6), DFR, (NADPH-dependent dihydro-flavonol reductase), LDOX (leucoanthocyanidin dioxygenase), UF3GT (UDP-Glc: flavonoid 3-Oglucosyltransferase), CCoAOMT1 (caffeoyl-CoA O-methyltransferase 1), CCR2 (cinnamoyl-CoA reductase 2), COMT2 (caffeic acid O-methyltransferase 2), C4H2 (cinnamate 4-hydroxylase 2), TAT1 (tyrosine aminotransferase 1), PAL1 (phenylalanine ammonialyase 1), PMT2 (putrescine N-methyltransferase 2), QPT2 (quinolinate phosphoribosyl-transferase 2), STR (strictosidine synthase) and TDC (tryphophan decarboxylase) [38,39,40,41,42,43,44,45,46]. The transcription factors are indicated in the colored boxes, including GSW1 (glandular trichome-specific WRKY 1), TCP14/15 (teosinte branched 1/cycloidea/proliferating cell factor 14/15), ORA (octadecanoid-derivative responsive AP2-domain protein), ALDH1 (aldehyde dehydrogenase 1), ERF15 (ethylene response factor 15), TASY (taxadiene synthase) and WND6A (wood-associated NAC domain transcription factor 6A).

Among the defense hormones salicylic acid (SA), ethylene (ET) and JAs, JAs are particularly renowned for their roles in inducing PSM production [35]. A role of JAs in PSM production in response to abiotic stresses (drought, temperature and ultraviolet light) is emerging, and the PSMs (e.g., glucosinolates and phytoalexins) induced by abiotic stresses at least partially overlap with those induced by biotic stresses [36,37]. Exogenously applied JA/MeJA or endogenous JA triggered by environmental cues could induce nearly all secondary metabolite biosynthetic pathways [28,36]. However, these compounds only represent a small number of JA-induced PSMs, given the species/lineage dependency of their induction. Although only a limited number of plant species have been investigated, JAs-induced compounds encompass all types of PSMs, including terpenoids, phenylpropanoids/phenols and N-/S-containing compounds (Figure 1).

We only briefly illustrated the great chemical diversity and species/lineage dependency of PSMs induced by the core JA signaling (Figure 1) (also, see reviews [25,26,27,47,48,49]). The core JA signaling is mediated by the bioactive JA-Ile, which is imported into the nucleus and perceived by the SCF^COI1-JAZ^ coreceptor complex, leading to the ubiquitination and proteasomal degradation of the JAZ transcriptional repressors. Subsequently, multiple JA-responsive transcription factors (TFs), preferably clade IIIe bHLH TFs such as MYC2, are relieved from JAZ-mediated repression, enabling them to interact with other proteins, such as MED25 and LEUNIG_ HOMOLOG (LUH), and in the subsequent recruitment of RNA polymerase II and HISTONE ACETYLTRANSFERASE1 (HAC1) to activate the expression of genes responsive to JAs [10,19,50,51,52,53]. Characterizations of TFs in the regulation of these JAs-induced PSMs confirm that MYC2 TFs act as the regulatory hub to elicit the production of diverse PSMs. Although this JA-Ile-mediated core signaling is conserved in land plants, the metabolic output under the control of these TFs is species-/lineage-specific (Figure 1). In several well-studied plant species such as *Arabidopsis thaliana*, *Artemisa annua*, *Taxus chinensis* and *Salvia miltiorrhiza*, MYC2 TFs are involved in inducing the biosynthesis of various terpenoids, which are the largest group of PSMs, comprising ~25,000 compounds. Among these terpenoids, linalool and (E)-β-caryophyllene; thujopsene and artemisinin; paclitaxel; tanshinones and salvianolic acids belong, respectively, to monotepenes (C10), sesquiterpenes (C15) and diterpenes (C20), which contain, respectively, two, three and four isopentenyl C5 units [54,55,56,57]. The JA-Ile signaling is also well-known to trigger the production of anthocyanin, which belongs to the group of phenylpropanoids/phenolics compounds. Plant phenolics are characterized by a hydroxyl functional group (phenyl group) on an aromatic ring, comprising simple phenols (e.g., SA and hydroxybenzoic acid derivatives) and polyphenols (e.g., anthocyanins, tannins, flavonoids and stilbenes) as well as miscellaneous phenolics (e.g., coumarins, resveratrol and lignins). Plant phenolics are the most broadly distributed secondary metabolites in plants, with approximately 10,000 compounds that take part in plant defense against pathogens and herbivores or in pollinator attraction [58]. More significantly, JA-Ile signaling is also involved in the induction of N-containing secondary metabolites such as alkaloids (e.g., nicotine and vinblastine), which have ~16,000 compounds and are prominent due to their dramatic physiological and psychological effects on humans. Furthermore, cyanogenic glycosides, another class of N-containing PSMs [59], have also been shown to be induced by JA-Ile signaling in *Lotus japonicas* [43]. Furthermore, JA-Ile is involved in the induction of S-containing compounds (including free aglycones and glycosides) such as glucosinolates (GLSs), which are responsible for the flavor and aroma of cruciferous vegetables and exhibit anticancer activity in humans. While alkaloids act in plant–herbivore interactions as toxins by disrupting DNA replication, protein synthesis and enzyme activity as well as neuronal signal transduction [60], GLSs are broken down into toxic cyanides upon cell disruption caused by attacks from herbivores or pathogens [61]. 

Characterization of TFs in the regulation of these JA-Ile-induced PSMs confirms that MYC2 TFs act as the regulatory hub to elicit the production of diverse PSMs. MYC2 TFs can directly regulate gene expression by binding the *cis*-element in the promoters of genes encoding enzymes in various pathways or by binding the same promoters but at different *cis*-elements with other TFs (e.g., AP2/ERFs) to synergistically regulate the biosynthesis of different PSMs, as illustrated in the production of distinct alkaloids in tobacco, tomato and *Catharanthus* [41,57,62,63,64]. Furthermore, MYC2 can directly control other downstream TFs such as the bHLH IRIDOID SYNTHESIS (BIS) TFs that regulate the monoterpene branch of the TIA pathway in *Catharanthus* [64,65,66] and the OCTADECANOID-DERIVATIVE RESPONSIVE CATHARANTHUS AP2-DOMAIN (ORCA) TFs that regulate other branches of the TIA pathway (Figure 1). Given this species/lineage specificity, studies in more diverse species would provide a basis to fully reveal the great chemical diversity and the underlying mechanism of PSMs induced by the relatively conserved JA-Ile signaling. 

## 3. Secondary Metabolism Induced by OPDA and CJ

Besides JA-Ile, other bioactive JAs have been generated in the processes of the biosynthesis and metabolism of JA, which have been extensively reviewed [2,3,4,5,6,7,8,9,10,11]. The first step of JA biosynthesis is the release of α-LA from chloroplast membranes by phospholipase A1 (PLA1), followed by the sequential actions of 13-lipoxygenase (13-LOX), allene oxide synthase (AOS) and allene oxide cyclase (AOC), leading to the formation of *cis*-(+)-OPDA in the chloroplast. Upon transport into peroxisomes, the cyclopentenone ring of OPDA is reduced by an OPDA reductase 3 (OPR3) to OPC8 (3-oxo-2-(2-pentenyl)-cyclopentane-1-octanoic acid), the carboxylic acid side chain of which is then shortened by three rounds of β-oxidation. After export to the cytosol, JA is conjugated with amino acids, mainly isoleucine, by jasmonoyl amino acid conjugate synthase (JAR1). Diverse JAs (derivatives of JA) are also produced by hydroxylation, carboxylation, decarboxylation and methylation sulfation [10].

**Figure 2:**
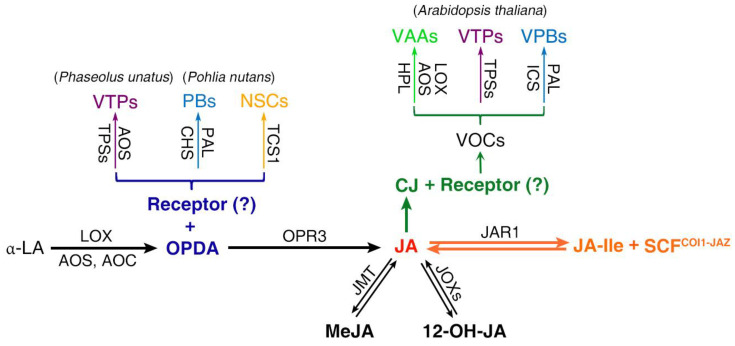
Plant secondary metabolites regulated by OPDA and CJ. α-LA undergoes the sequential actions of LOX, AOS and AOC, leading to the formation of OPDA. OPDA is then reduced by OPR3 and shortened by three rounds of β-oxidation to produce JA. JAs include CJ, MeJA, 12-OH-JA and JA-Ile. While JA-Ile activates the core JA signaling pathway by binding with its coreceptor SCF^COI1-JAZ^, OPDA and CJ activate distinct signaling independent of JA-Ile; however, the existence and identity of their cognate receptors remain unclear. The main catalyzing enzymes are indicated: JMT (jasmonic acid carboxyl methyltransferase), JOXs (jasmonate-induced oxygenases), JAR1 (jasmonoyl-isoleucine synthetase), CHS (chalcone synthase), TCS1 (tea caffeine synthase 1), HPL (hydroperoxide lyase) and ICS (isochorismate synthase). The types of PSMs are shown as VTPs (volatile terpenoids), PBs (phenylpropanoids /benzenoids), NSCs (nitrogen-/sulfur-containing compounds), VAAs (volatile alcohols/aldehydes) and VPBs (volatile phenylpropanoids/benzenoids).

Signaling independent of JA-Ile has been established for OPDA, a key biosynthetic precursor of JA [67]. OPDA is analogous to mammalian prostaglandins (PGs) in terms of both molecular structure and physiological functions, such as wound healing and regulation of reproductive systems [68]. This notion has also been supported by biomedicinal studies showing that OPDA represses breast cancer cell proliferation [69], modulates inflammation induced by lipopolysaccharide in mouse brain cells [70] and suppresses H_2_O_2_-induced cytotoxicity in human neuroblastoma cells [71]. The role of OPDA in promoting plant stress resistance and growth arrest in a JA-Ile-dependent or JA-Ile-independent manner has been reviewed recently [72]. Although exogenous application or overexpression of OPDA biosynthetic enzymes was important in identifying OPDA-specific responses, such OPDA-boosting approaches are inherently compromised by the conversion of OPDA to other JAs including JA-Ile. Exploiting *Arabidopsis* mutants that failed to convert OPDA to JA-Ile, such as *opr3*, *opr2opr3* and *opr3coi1*, to uncouple the JA-Ile and OPDA signaling has provided evidence to support JA-Ile-independent signaling for OPDA. In contrast to vascular plants, nonvascular plants, such as Marchantia and *Physcomitrium patens*, do not produce JA or its derivatives but do produce OPDA and dn-OPDA, which is derived from hexadecatrienoic acid and is two carbons shorter than OPDA in the carboxylic acid side chain. COI1-JAZ-dependent signaling is conserved but uses dn-OPDA as the ligands in Bryophytes, thus rendering them excellent models for exploring both OPDA-specific responses and the evolution of OPDA and JA-Ile signaling [17,73,74,75,76]. Although the involvement of OPDA in the stress response has been confirmed, the role of OPDA in eliciting PSMs is just emerging (Figure 2). Exogenous application of OPDA and JA mediated different patterns of volatile production in lima beans. OPDA could promote the biosynthesis of the diterpenoid-derived 4,8,12-trimethyltrideca-1,3,7,11-tetraene (TMTT) and even more complex patterns of additional volatiles under high OPDA concentrations [77]. Furthermore, the phytoalexin production in the *hebiba* rice mutant that is deficient in oxylipins was able to be restored through treatment with OPDA rather than JA-Ile [78]. These results illustrate that OPDA regulates the production of PSMs independent of JA-Ile signaling. Widely targeted metabolomics analyses of the OPDA-induced metabolome in Antarctic moss (*Pohlia nutans*) show that a total of 82 metabolites were significantly changed by OPDA treatment, including alkaloids, phenolic acids, flavonoids and amino acids and derivatives [79]. However, whether the induction of these PSMs by OPDA is independent of dn-OPDA, and if so, its underlying mechanism, awaits more comprehensive analyses.

CJ is a volatile organic compound (VOC) belonging to JAs that is emitted constitutively from the flowers or leaves of several plant species [70] or released upon herbivory [80,81,82], the application of insect saliva [83,84,85], treatment with JA [86] or inoculation with nitrogen-fixing rhizobacteria [87]. It has been proposed that CJ is produced by dehydration and decarbonation of JA in the peroxisome [86], although an origin from *iso*-OPDA has also been proposed [88]. However, convincing evidence of CJ biosynthesis and signaling in plant cells is still lacking. The capacity of CJ to elicit plant defense has been investigated in *Arabidopsis* [89], *Brassica* cultivars [90], soybean [91,92], potato [93], tomato [94], cotton [95] and sweet pepper [96] as well as cereals [97,98,99,100]. CJ has been shown to enable plants to repel/deter or be primed to defend against many insect pests including aphid species, *Myzus persicae* (Sulzer), *Aulacorthum solani* (Kalt), *Sitobion avenae* (Fabricius), *Nasonovia ribis-nigri* (Mosley), *Aphis gossypii* (Glover), *Macrosiphum euphorbiae* (Thomas), *Lipaphis erysimi* (Kaltennbach) (Hemiptera: Aphididae) [80,89,93,95,96,97], cereal leaf beetle, *Oulema melanopus* (Linnaeus) (Coleoptera: Chrysomelidae) [99], the leafhopper *Cicadulina storeyi* (China) (Hemiptera: Cicadellidae) [98], *Frankliniella occidentalis* (Pergande) (Thysanoptera: Thripidae) [101,102] and the beet armyworm (Lepidoptera: Noctuidae) [94], mostly in the laboratory, and two aphid species, *Phorodon humuli* (Schrank) and *S. avenae*, in the field [80,97,103]. Intriguingly, CJ is involved in plant–herbivore–natural enemy tritrophic interaction by attracting the predators/parasites of herbivores such as *Coccinella septempunctata* (Linnaeus) (Coleoptera: Coccinellidae), *Aphidius ervi* (Haliday) (Hymenoptera: Braconidae) and *Telenomus podisi* (Ashmead) (Hymenoptera: Scelionidae) [80,89,91,96] under laboratory conditions and scelionid wasps under field conditions [92]. Furthermore, CJ treatment appears to attract natural enemies for generalist but not specialist herbivores [80,89,95,96,97].

Plants synthesize and release a great diversity of VOCs to communicate with other organisms that are important for their reproduction and defense [104,105]. CJ induces the emission of a diverse mixture of VOCs (Figure 2 and Table 1) that encompass all types of PSMs including terpenoids, phenylpropanoids and fatty acid derivatives (Figure 1). GC coupled with electroantennography (GC-EAG) analysis and olfactometer bioassay has shown that CJ-induced VOCs such as methyl SA (MeSA), (E)-4,8-dimethyl-1,3,7-nonatriene (DMNT) and TMTT could mediate plant–insect interactions. Similar to JA-Ile, CJ-induced VOC profiles are also lineage-specific; together with the fact that generalist and specialist aphids responded differentially to CJ [89], this supports the idea that CJ may act as a signaling molecule in addition to a semiochemical. CJ primes *Zea mays* for enhanced production of defensive VOCs (e.g., caryophyllene, bergamotene, farnesene and DMNT) against the leafhopper, *C. storeyi* [98]. CJ treatment makes a range of brassica cultivars less attractive to and less suitable for *M. persicae* but more attractive to *Diaeretiella rapae* [90]. Consistently, CJ can be perceived by *A. thaliana* plants to induce a discrete and distinctive suite of genes by signaling independent of JA-Ile [106]. Noticeably, genes encoding TGA2, TGA5 transcription factors and TGA6 as well as *CYP81D11*, *OPR1* and *GST25* are induced by OPDA and phytoprostanes (structurally related electrophilic cyclopentenones), CJ and xenobiotics (e.g., 2,3,5-Triiodobenzoic acid, TIBA) [106,107,108], implying that the signaling pathways of these chemicals intersect. 

## 4. JAs Are Involved in Integrating and Orchestrating Primary and Secondary Metabolism

Plants have evolved constitutive and inducible defense strategies against herbivores and pathogens. In inducible defenses, immune signaling and response are activated after the perception of signals derived from herbivores by pattern-recognition receptors (PRRs) at the cell surface and nucleotide-binding leucine-rich repeat proteins (NLRs) mostly inside the cell. Among these outputs, the accumulation of plant defense hormones, including SA, JA and ET both locally (in infected sites) and systemically (in uninfected sites), is a key event. However, the activation of the defense response is usually associated with suppressed plant growth, and this antagonistic relationship is often referred to as the ‘growth–defense tradeoff’. Two hypotheses have been put forward to explain the mechanisms of the growth–defense tradeoff. Since the defense network imposes a substantial demand for resources, an inverse relationship between defense and growth may contribute to the resource (e.g., carbon and nitrogen) redistribution between photosynthesis and growth and to the secretion of defense proteins and production of defense compounds (predominantly PSMs). Alternatively, reprogramming of gene expression and resource redistribution may be an active and hardwired plant strategy to regulate growth–defense tradeoffs [44,109].

Intriguingly, the constitutive activation of JA responses confers upon plants reduced growth and increased content of PSMs, whereas *A. thaliana* and rice (*O. sativa* L.) *coi1* mutants that are deficient in the coreceptor of JA-Ile showed enhanced growth [110]. Consistently, the *atjat1* mutants deficient in the transporter-mediating nuclear entry of JA-Ile showed compromised resistance to the necrotrophic fungal pathogen *Botrytis cinerea* but enhanced plant growth [111]. Progressive mutation of *JAZs* showed that the strength of the core JA signaling was positively correlated with defense but inversely with growth and fertility [112]. A prominent role of JA-Ile signaling in coordinating growth–defense antagonism thus provides an attractive opportunity to unravel the mechanisms underlying growth–defense tradeoffs. 

**Figure 3:**
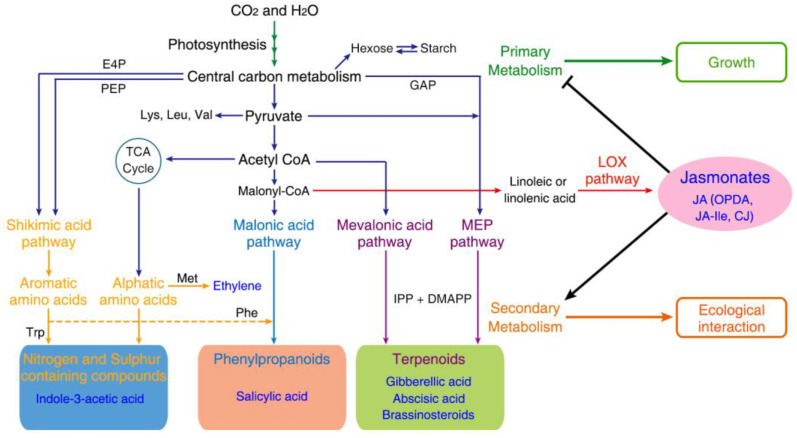
Roles of jasmonates in coordinating secondary with primary metabolism. Primary metabolism (dark-blue lines) starts with routes of photosynthesis and central carbon metabolism. The diverse PSMs belonging to nitrogen-/sulfur-containing compounds, phenylpropanoids and terpenoids enable plants to adapt to biotic and abiotic environments by mediating ecological interaction. Plant hormones auxin, SA, GAs, BRs, ET and JAs are shown in their respective PSM families. JA and bioactive JA-Ile, OPDA and CJ are shown to promote the production of PSMs for defense and suppress the primary metabolism for growth/development. TCA (tricarboxylic acid), MEP (methylerythritol 4-phosphate), shikimic acid, malonic acid and mevalonic acid pathways and main catalyzing enzymes E4P (erythrose-4-phosphate), PEP (phosphoenolpyruvate) and GAP (glyceraldehyde 3-phosphate) are shown.

Secondary metabolites use primary metabolites as the main building blocks (Figure 3), as illustrated by the fact that many amino acids act as the bridge between primary and secondary metabolism by functioning as the precursors both of proteins and many PSMs [113]. The composing isopentenyl pyrophosphate unit of terpenoids is generated by photosynthetic activities, the 2-C-methyl-D-erythritol 4-phosphate (MEP) pathway as well as the mevalonate (MVA) pathway. Shikimic acid is the precursor of the shikimate pathway and is derived from erythrose 4-phosphate (pentose phosphate pathway) and phosphoenolpyruvate (glycolytic pathway) [114]. The end product of the shikimate pathway, chorismite, acts as the precursor of tyrosine, tryptophan, salicylate, phenylalanine, phylloquinone and folate [115]. Phenylalanine is a precursor for phenolics such as flavonoids, lignin, condensed tannins, betalain pigments and quinones, while tryptophan acts as a precursor of alkaloids, indole glucosinolates and phytoalexins. In alkaloids except pseudoalkaloids (e.g., capsaicin, solanidine and caffeine) that are not derived from amino acids, true alkaloids (e.g., nicotine, quinine, atropine and morphine) are produced from amino acids and share a N-containing heterocyclic ring [116], while proto-alkaloids (e.g., yohimbine, mescaline and hordenine) are mainly derived from L-tryptophan and L-tyrosine and contain a nitrogen atom that is not part of the heterocyclic ring. 

The accumulated data establish JAs as a regulatory hub that shifts the central metabolism from the production of growth-stimulating metabolites to the production of protective compounds, predominantly precursors of PSMs [117]. A decreased level of UDP-glucose in an *Arabidopsis aos* mutant supports the role of JAs in regulating the sucrose synthase pathway and the sink/source metabolic status [112,118]. A decreased level of P-gluconate in plants with depleted JAs and an induced oxidative pentose phosphate pathway in multiple-*jaz* *Arabidopsis* mutant with enhanced JA-Ile signaling [112] indicates that JAs are involved in regulating the oxidative pentose phosphate pathway. Furthermore, JAs are also involved in the regulation of the tricarboxylic acid (TCA) cycle, metabolites of which are intermediates of the central metabolism and branching points for the production of PSMs. In *Arabidopsis* plants with depleted JAs, the content of 3-phosphoglyceric acid, dihydroxyacetone phosphate, glyceraldehyde 3-phosphate and phosphoenolpyruvates that are glycolytic intermediates; oxaloacetate; and 2-oxoglutarate derived from the TCA cycle were reduced. However, the levels of citrate, aconitate, isocitrate, malate and fumarate, metabolites of the TCA cycle, were significantly increased [119,120]. The contents of aromatic and branched chain amino acids were reduced in Arabidopsis plants deficient in JAs [120] but transiently increased in *Nicotiana. tabacum* leaves treated exogenously with JA [121]. 

Collectively, these findings provide evidence to support the roles of JAs in orchestrating growth–defense tradeoffs by shifting the central metabolism from producing growth-simulating metabolites to producing protective compounds that act predominantly as the precursors of PSMs. Given that the growth-promoting plant hormones auxin, gibberellic acid (GA) and brassinosteroids (BRs) also belong to PSMs (Figure 3), competition for substrates common to these phytohormones and other PSMs could underlie JAs-regulated growth–defense tradeoffs; however, this mechanism remains largely unexplored. Significantly, the cross-talking of JA-Ile signaling with the signaling of these growth-promoting hormones, particularly GA [110,112], has been well recognized as playing a vital role.

## 5. Conclusions, Perspectives and Future Directions

The preeminent role of JA-Ile signaling in the induction of a great diversity of PSMs has been well established in flowering plants, particularly in plants with high pharmaceutical values. Combinatorial or even modular control of the catalyzing enzymes by multiple transcription factors provides a mechanistic explanation for the diversity and lineage specificity in the production of PSMs induced by JAs. Although JA-Ile singling is recognized as playing a major role in the production of JAs-induced PSMs, increasing evidence supports the idea that through signaling independent of JA-Ile, OPDA can also induce the production of diverse PSMs, while CJ can induce the production of VOCs. Furthermore, JAs are involved in coordinating secondary with primary metabolism, which plays essential roles in the regulation of growth–defense tradeoffs. 

Currently, plant secondary metabolism and primary metabolism are recognized to be equally important and form a dynamic metabolic network enabling plants to survive, grow and reproduce in diverse and everchanging environments [29,110]. The renowned roles of JAs in triggering a great diversity of PSMs in a lineage-specific manner [9,25,28] provide a unique opportunity to address questions such as the proportion of PSMs controlled by JAs in diverse plant species and how transcriptional modules in the core JA signaling pathway determine the great chemical diversity of PSMs, as well as the mechanisms underlying their roles in the adaptation to their environments. Of particular interest is the mechanism of JAs in the coordination of plant growth–defense tradeoffs by steering central metabolism between metabolic pathways for growth and development and specialized metabolism for defense or other ecological interactions. Although evidence is emerging for roles of OPDA in promoting the production of PSMs and of CJ in triggering the production of VOCs, their signaling pathways independent of JA-Ile await elucidation. In addition, the regulatory roles of JAs in PSM production in beneficial interactions between plants and pollinators, fructivores/seed-dispersers and natural enemies are intriguing and should be further explored. A key role of CJ and its elicited VOCs in plant–herbivore–natural enemy tritrophic interaction has been well established in *Arabidopsis* as well as many crops; however, the biosynthetic and signaling pathways of CJ remain largely unclear. Due to their direct damage to crops and indirect damage as vectors of plant viruses, aphids are major crop pests worldwide. The CJ-regulated plant–aphid–natural enemy system would provide new insights into the tritrophic interaction based multiple-trophic interactions that are important for biological community assembly. Additionally, many PSMs elicited by JAs are important for their pharmaceutical value (e.g., vinblastine, artemisinin, taxol, GLSs and ginsenoside) in a range of medicinal plant species [47]. Thus, this fundamental mechanistic understanding of plant secondary metabolism induced by JAs will facilitate the design of environmentally friendly crops and medicinal or aromatic plants. Moreover, the synthetic biology approach offers a novel path to improve the production of secondary metabolites, including JAs (e.g., OPDA and CJ), in plant and microbial cells. 

## Figures and Tables

**Table 1:** The VOCs induced by CJ.

Plant Species	CJ-Induced VOCs	Refs.
*Arabidopsis* *thaliana*	Fatty acid derivatives: (Z)-3-hexen-1-ol, 1-octen-3-ol Terpenoids: 6-methyl-5-hepten-2-one Phenylpropanoids/benzenoids: ethylbenzene, 4-ethyltoluene Specific compounds: heptyl isothiocyanate	[89]
*Glycine max*	Terpenoids: camphene, myrcene, (E)-ocimene, TMTT Phenylpropanoids/benzenoids: MeSA	[91]
*Capsicum annum*	Fatty acid derivatives: (Z)-3-hexenyl acetate, (Z)-3-hexenyl butyrate Terpenoids: sabinene, myrcene, geranylacetone, 6-methyl-5-hepten-2-one	[96]
*Gossypium hirsutum*	Fatty acid derivatives: (Z)-3-hexenyl acetate Terpenoids: DMNT, TMTT Phenylpropanoids/benzenoids: MeSA	[95]
*Zea mays*	Terpenoids: (E)-(1R,9S)-caryophyllene, (E)-a-bergamotene, (E)-β-farnesene, DMNT	[98]
*Solanum lycopersicon*	Fatty acid derivatives: (E)-2-hexenal, (Z)-3-hexen-1-yl butyrate, nonanal, decanal Terpenoids: α-pinene, α-copaene, (E)-β-farnesene, DMNT, TMTT Phenylpropanoids/benzenoids: MeBA, MeSA Aromatic compounds: indole	[94]
*Brassica napus*, *B. rapa* and *B. oleracea*	Fatty acid derivatives: 2-ethyl-1-hexanol, 1-octanol, 2-butyl-1-octanol, nonanal, decanal, *cis*-3-hexenyl acetate, dihydrojasmone, *cis*-jasmone Terpenoids: citronellol, p-cymen-7-ol, α-elemene, α-curcumene, (E, E)-β-farnesene, DMNT Phenylpropanoids/benzenoids: MeSA, benzothiazole Specific compounds: methyl isothiocyanate, benzyl nitrile Aliphatic hydrocarbons: dodecane, (E)-3-tetradecene	[90]
*Triticum aestivum* L. and *Hordeum vulgare* L.	Fatty acid derivatives: (Z)-3-hexanal, (Z)-3-hexanol, (Z)-3-hexanyl acetate Terpenoids: (Z)-β-ocimene, linalool, β-caryophyllene, (E)-β–farnesene Aromatic compounds: indole	[99]
